# Cognitive Performance following Carotid Endarterectomy or Stenting in Asymptomatic Patients with Severe ICA Stenosis

**DOI:** 10.1155/2013/342571

**Published:** 2013-12-21

**Authors:** Livio Picchetto, Gianfranco Spalletta, Barbara Casolla, Claudia Cacciari, Michele Cavallari, Cristiano Fantozzi, Alessandro Ciuffoli, Maurizia Rasura, Francesca Imperiale, Giuliano Sette, Carlo Caltagirone, Maurizio Taurino, Francesco Orzi

**Affiliations:** ^1^Department of Neuroscience, Mental Health and Sensory Organs (NESMOS), University of Rome “La Sapienza”, Sant'Andrea Hospital, Via di Grottarossa 1035-1039, 00189 Rome, Italy; ^2^Fondazione Santa Lucia, Instituto di Ricovero e Cura a Carattere Scientifico (IRCCS) and Memory Clinic, V. Ardeatina 306, 00179 Rome, Italy; ^3^Department of Vascular Surgery, Sant'Andrea Hospital, University of Rome “La Sapienza”, Via di Grottarossa 1035-1039, 00189 Rome, Italy; ^4^Department of Neurology and Psychiatry (Parkinson's Centre) and Research Centre of Social Diseases (CIMS), University “La Sapienza”, Viale dell'Università 20, 00185 Rome, Italy

## Abstract

*Background*. Endarterectomy (CEA) or stenting (CAS) of a stenotic carotid artery is currently undertaken to reduce stroke risk. In addition removal of the arterial narrowing has been hypothesized to improve cerebral hemodynamics and provide benefits in cognitive functions, by supposedly resolving a “hypoperfusion” condition. *Methods*. In this study we sought to test whether resolution of a carotid stenosis is followed by measurable changes in cognitive functions in 22 subjects with “asymptomatic” stenosis. *Results*. A main finding of the study was the statistically significant pre-post difference observed in the performance of phonological verbal fluency and Rey's 15-word immediate recall. Remarkably, there was a significant interaction between phonological verbal fluency performance and side of the carotid intervention, as the improvement in the verbal performance, a typical “lateralized” skill, was associated with resolution of the left carotid stenosis. *Conclusion*. The results reflect a substantial equivalence of the overall performance at the before- and after- CEA or CAS tests. In two domains, however, the postintervention performance resulted improved. The findings support the hypothesis that recanalization of a stenotic carotid could improve brain functions by resolving hypothetical “hypoperfusion” states, associated with the narrowing of the vessels.

## 1. Introduction

Asymptomatic patients with substantial (i.e., 60%–90%) carotid artery narrowing, but no recent neurological symptoms, are at increased long-term risk of ischemic stroke, especially in areas of the brain supplied by the artery. Endarterectomy (CEA) or stenting (CAS) of the carotid artery removes the arterial narrowing, but the procedure itself causes immediate risk of stroke or death. It is object of a long-lasting debate to establish whether the risk overcomes what seems to be a substantial reduction of nonperioperative stroke over a 10-year period following CEA [1]. Improvements in stroke prevention obtained by medical treatment alone and care, in the last few years, seem to support conservative approaches in asymptomatic subjects [2, 3]. The debate is still ongoing [4].

A side aspect, consistently dismissed in clinical settings, concerns the notion that CEA or CAS may improve brain functions by resolving a hypothetical “hypoperfusion” state, associated with narrowing of the vessels [5–7]. The hypothesis has been probably underestimated for long time, as supportive data were essentially of anecdotal nature. A number of case reports have shown improvement in cognitive impairment following CEA or extracranial-intracranial bypass [8–10]. The improvement seems to be enhanced in patients with chronic internal artery occlusion and cerebral ischemia [11]. Most of the reported data, however, miss neuropsychological assessment or refer to a variety of different clinical conditions, which include both symptomatic and asymptomatic carotid stenoses. The interpretation of the results remains uncertain. Recent data, mostly based on sophisticated semiquantitative neuroimaging, seem to advocate a revival of the hypothesis [12]. Thus, in addition to removing local causes of downstream altered circulation, CEA or CAS would improve cerebral hemodynamics and provide benefits in cognitive functions. A mechanism for the improved hemodynamics associated with carotid recanalization might rely on the enhancement of collateral circulation through the Circle of Willis [1]. Chmayssani et al. demonstrated that carotid artery re-canalization restores the cerebral vasoreactivity impairment and the altered fMRI patterns associated with the narrowing of the vessel [13]. The finding seems to support the hypothesis that cells in the potentially “chronically hypoperfused” hemisphere persist in a dysfunctional state that could be reversed by resolution of the carotid stenosis and restoration of a normal flow in the vessel. This hypothesis is very difficult to test, as both the “hypoperfusion” and “dysfunction” concepts lack at present an operational definition.

In this study we sought to address the issue by simply trying to test whether resolution of a carotid stenosis is followed by measurable changes in cognitive functions. Transcranial Doppler (TCD) measurements were carried out in a few subjects to document the hypothesized improved vasoreactivity associated with CEA or CAS. In order to limit potential confounding variables associated with recovery from a previous stroke we included in the study only subjects with “asymptomatic” stenosis, that is, subjects with no clinical history of stroke or TIA.

## 2. Methods

### 2.1. Patients

Subjects included in the study were consecutive patients referred (from November 2009 to December 2011) to the vascular surgery unit, because of carotid stenosis judged to be eligible for CEA or CAS. All the patients were tested for carotid stenosis within a check-up that aimed to assess the risk of cerebrovascular diseases, as advised by the family physician or cardiologist. In all the cases the stenosis was asymptomatic (without history of previous stroke or TIA). Carotid artery stenosis was documented by neck duplex ultrasound, CT angiography, or MR angiography.

Exclusion criteria were life expectancy <1 year, presence of dementia or psychiatric comorbidity (according to the DSM-IV criteria), and history of recent (within 3 months) ischemic stroke (assessed either on clinical or neuroimaging bases). Patients with mild white matter abnormalities or small gliotic lesions on MRI were included.

All the subjects underwent MRI, including diffusion-weighted imaging, before (1–3 days) and after (1–4 days) treatment. MRI data were examined by a neuroradiologist blinded to treatment and outcome.

All the subjects gave their written informed consent to participate in the study. The study was approved by the local ethics committee.

### 2.2. Neurological, Neuropsychological, and Psychiatric-Behavioral Assessments

Neurological examination, including scoring by NIHSS, was performed just before and 1 day after CEA or CAS.

The neuropsychological assessment was carried out before (1–3 days) and after (3 months) CEA or CAS. The assessment consisted of the mental deterioration battery [14] and modified Wisconsin card sorting tests [15]. The mental deterioration battery is a standardized and validated neuropsychological tool for assessing elaboration of verbal material and constructional praxis. It includes 5 tests: Rey's 15-word immediate or delayed recall, copying drawings or copying drawings with landmarks, and phonological verbal fluency. The Wisconsin tests allow us to assess set-shifting or cognitive flexibility, that is, the ability to alter a behavioural response mode in the face of changing contingencies. The tool comprises the following 2 tests: Wisconsin card sorting test for categories or perseverative errors.

In order to limit practice effects in the follow-up assessment, wherever possible, parallel forms of the tests were used, that is, Rey's 15-word immediate or delayed recall and phonological verbal fluency. Scores were adjusted for age and educational level.

A psychiatric and behavioural evaluation was carried out contextually with the neuropsychological assessment. Depressive symptom severity was evaluated by the 21-item Beck depression inventory [16] and the 17-item Hamilton depression rating scale [17], both including psychic and somatic subscales that contribute to the total score. Severity of anxiety was measured by the Hamilton anxiety rating scale [18].

### 2.3. CEA or CAS Interventions

The choice between CEA and CAS was based on the presence of comorbidities, vessel anatomy, or characteristics and location of the plaque, according to the CREST criteria [19]. Operative procedures were performed either under general or local anaesthesia, according to the type of intervention and comorbidities. All patients were anticoagulated with heparin at the time of the intervention. Patients were given perioperative aspirin or low-molecular-weight dextran. Specifically, CAS was performed in patients presenting high surgical risk factors, including advanced age and cardiac and pulmonary diseases, or in patients in whom the stenosis was inaccessible to CEA. The procedure was carried out using self-expanding stents, following application of distal filters or proximal balloon protection devices, via puncture of the right or left femoral artery. Atropine (0.5 to 1 mg) was given intravenously to most of the patients to reduce bradycardia and hypotension, potentially associated with carotid dilatation.

### 2.4. TCD with Hypercapnic Stimulation

Vasomotor reactivity was evaluated in 8 subjects, before (1–6 days) and after (about 3 months) revascularization, by means of transcranial Doppler ultrasonography (Multi-Dop X/TCD instrument, DWL Elektronische Systeme GmbH) and hypercapnic stimulation. The testing was carried out in a supine resting state with eyes closed. All the subjects had abstained from smoking, alcohol, or caffeine-containing beverages for at least 12 hours before the assessment. One dual 2 MHz transducer fitted on a headband and placed on the temporal bone window was used to obtain continuous measurements. TCD monitoring of the ipsilateral MCA was carried out at an insonation depth of 50 to 56 mm as described in previous studies [20]. After 3 minutes of baseline measurement, subjects breathed 5% CO_2_ air solution via facemask for 3 minutes. A vasomotor reactivity index (VMRI) was computed as the ratio between percentage increments in blood velocity and arterial pCO_2_ (mmHg) [21]. The index has been largely employed to assess vasomotor reactivity [22–24].

### 2.5. Statistical Analysis

Data were analyzed by means of the two-way ANOVA. The analysis was followed by Bonferroni's post hoc correction for multiple comparisons. Individual cognitive scores were considered as dependent variables. The factors were time (pre-post CEA or CAS) and side (left or right carotid). The test was carried out independently for the sets of data pertaining each of the domains tested.

## 3. Results

The study population consisted of 22 patients, 14 males and 8 females. CEA was carried out in 10 and CAS in 12 of the subjects. There was no serious perioperative complication. One subject had a transient mild anaemia, and a second patient had a femoral hematoma. Sociodemographic and clinical characteristics are showed in Table 1. All the enrolled patients presented severe (>70%) carotid stenosis. The stenosis was greater than 80% in 17 subjects (81%). A contralateral stenosis was found in 7 (32%) of the patients and only 4 (18%) subjects had contralateral stenosis greater than 50%.

The CEA/CAS interventions were carried out on the right carotid in 12 (55%) and on the left carotid in 10 (45%) of the subjects.

Comparison of DWI carried out before and after CEA or CAS did not show any relevant difference, except for 3 patients who presented a few (from 1 to 4) small (<5 mm diameter) ipsilateral areas of altered signal 1 to 4 days after endovascular intervention (CAS), compatible with silent ischemic lesions (NIHSS = 0).

The vasoreactivity index was computed in a subsample of 8 subjects, before and after CEA or CAS. In all the cases there was, as expected, a substantial difference between the two measurements, as evidence of an improved vasoreactivity following the intervention (Table 2).

**Figure 1:**
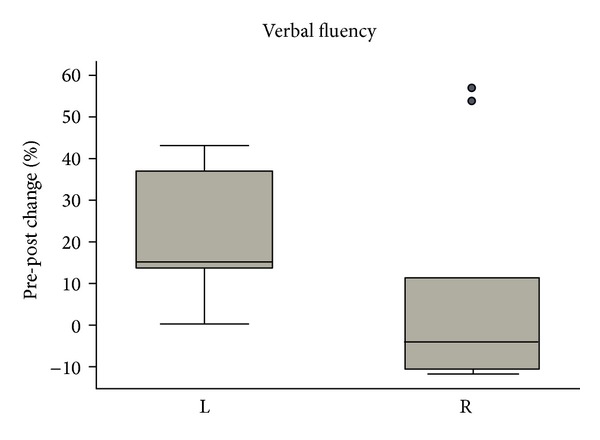
Correlation between phonological verbal fluency performance and side of the carotid intervention (*P* < 0.05) Side. L: left; R: right.

All the patients underwent the neuropsychological and behavioural assessments before and after CEA or CAS. The postintervention performance appeared superior to the preintervention one in 2 of the 7 tests. Thus, a statistically significant pre-post difference was observed in the phonological verbal fluency (*F* = 9.348; *P* < 0.007) and in Rey's 15-word immediate recall (*F* = 7.949; *P* = 0.01) (Table 3). Remarkably, there was a significant interaction between the phonological verbal fluency performance and side of the carotid intervention (*F* = 5.107; *P* = 0.037) (Figure 1).

The psychiatric-behavioural assessment was conducted to evaluate the presence and severity of behavioural symptoms potentially relevant in affecting the results of the neuropsychological assessment. We observed no difference between the pre- and postscoring in any of the psychiatric-behavioural evaluations carried out (Table 4).

## 4. Discussion

In this study we evaluated the potential cognitive impact of CEA or CAS by performing a number of neuropsychological tests before and after the intervention. In order to avoid confounding variables associated with recovery from a previous stroke or associated with progression of preexisting cerebrovascular lesions, we enrolled subjects with asymptomatic stenosis. All the included subjects were, therefore, patients who had been referred to the vascular surgeon for primary prevention. We performed MRI before and after the intervention in order to account for “silent” lesions (present before the enrollment or caused by the intervention itself) potentially relevant in influencing the performance at the tests. We also carried out a psychopathological evaluation in order to account for potential confounding behavioral variables. The simple study design allowed a typical pre-post statistical analysis, effective in disclosing cognitive benefits or drawbacks of the recanalization procedures. The first testing was carried out 1–3 days before and the second one 3 months following CEA or CAS.

The results of the study reflect a substantial equivalence of the overall performance at the before- and after-CEA or CAS tests. In two domains, however, the second performance was definitely different from the first one. A statistically significant pre-post difference was in fact observed in the phonological verbal fluency and in the Rey's 15-word immediate tests. The difference in phonological verbal fluency test remained significant following adjustment of the critical *P* values in order to account for the seven independent pre-post comparisons (Bonferroni's correction for multiple comparisons). The two tests refer to cognitive skill which are considered highly “lateralized.” The side of the intervention seems to be relevant to the improvement, as CEA or CAS of the left carotid accounted, in our study, for most of the effects on verbal fluency. All the subjects were right handed.

Our data are consistent with the findings by Chmayssani et al. [13]. Our findings do in fact support the hypothesis that a state of unilateral impaired cerebral hemodynamics is sufficient to cause subtle cerebral dysfunctions, which are potentially reversible following removal of the carotid stenosis. Our data do not obviously prove the causal relationship between carotid stenosis and altered hemodynamics, but the observation of an improved vasoreactivity index (as evaluated by TCD and hypercapnia) in all the subjects in whom the test was performed is also consistent with the hypothesis of an impaired cerebral hemodynamics associated with the carotid stenosis. The hemodynamic assessment, however, was carried out just in 8 subjects. Testing for a causal correlation between the hemodynamic changes and the neuropsychological improvement was behind the scope of the study. The sample size to have sufficient statistical power would presumably be too large. It is reasonable to speculate, however, that the hypothesized “hypoperfusion” essentially consists in an impaired functional hyperaemia, to cause inadequate oxygen and energy substrate supply only when the tissue energy demand is increased. Thus, it is a hypothesized that the carotid stenosis contrasts the required increment of blood flow under conditions of increased energy demand.

Previous studies have reported conflicting effects of CEA or CAS on cognition [25]. A number of methodological (statistics, follow-up schedule, presence of a control group, and type of neuropsychological assessment) and patient-related (symptomatic or asymptomatic patients, severity of carotid stenosis) variables make a comparison of the different studies unlikely meaningful. Most of the studies were carried out in patients with symptomatic stenosis. In these patients the evolution of the pre-existing stroke may constitute a remarkable confounding variable in the interpretation of the neuropsychological assessment [8]. A study by Grunwald et al., in which cognitive assessment was carried out following CAS in asymptomatic patients, obtained results consistent with ours [26]. Other sources of uncertainties probably rely on study designs. For instance, a few studies were based on the comparison between patients and controls, being the two groups inevitably inhomogeneous [27, 28].

In this study we sought to minimize the variables by focusing on asymptomatic subjects and by exploiting a before-after study design. This approach accounts for confounding variables, such as possible subtle cognitive impairments that have been shown to be present in subjects with “asymptomatic” stenosis, when properly tested [29].

This study has, however, a number of limitations. The small sample size is probably the major restraint, although the paired nature of the study design has added enough power to the statistical approach. Furthermore, the nature of the study could not allow a blind design, and the “after” assessment could be biased by expectation or learning effect. We tried, however, to account for this potential bias by using parallel form of the tests validated to limit “practice effects” and by performing psychopathological tests that aimed to detect depressive or anxiety symptoms, which could affect the neuropsychological performance.

In conclusion, our results reflect a substantial equivalence of the overall performance at the before- and after-CEA or CAS tests. In two domains, however, the postintervention performance which resulted improved. We cannot exclude that the apparent improvement reflects other variables associated, for instance, with learning or release of anxiety. A confidence, however, in the neurovascular fundament of the observed improvement arises from the significant interaction between the phonological verbal fluency performance and side of the carotid intervention, suggesting that a supposedly “lateralized” function benefits mostly from resolution of a homolateral carotid stenosis. Our findings do, therefore, support the hypothesis that recanalization of stenotic carotid improves brain functions by resolving hypothetical “hypoperfusion” states, associated with the narrowing of the vessels.

## Figures and Tables

**Table 1:** Descriptive characteristics (*n* = 22).

Demographics	
Age, y	70 ± 7
Male	14 ± 64
Education, y	11 ± 6

Risk factors	
Diabetes	4 (18%)
Hypertension	18 (82%)
Atrial fibrillation	3 (14%)
Hyperlipemia	7 (32%)
Cardiac failure	2 (9%)
Current smokers	5 (23%)

Carotid stenosis features	
Right >70%	12 (55%)
Left >70%	10 (45%)
Contralateral stenosis	7 (32%)

Revascularization procedure	
CAS	12 (55%)
CEA	10 (45%)

Data are means ± SD, or number of cases and percentage in brackets. CAS: carotid artery stenting; CEA: carotid endoarterectomy.

**Table 2:** Cerebral vasomotor reactivity index (VMRI).

	PRE	POST
CEA	2.3 ± 1.3	3.9 ± 0.7*
CAS	3.6 ± 1.5	4.0 ± 1.5*

Data are means ± SD for 8 of the 22 subjects included; *P < 0.01; paired *t* test. CEA: carotid endoarterectomy; CAS: carotid artery stenting.

**Table 3:** Neuropsychological performance before and after CEA or CAS.

Rey auditory verbal learning test, immediate recall	32.9 ± 9.4	37.2 ± 11.2*
Rey auditory verbal learning test, delayed recall	6.5 ± 2.5	7.4 ± 3.5
Phonological verbal fluency	28.9 ± 11.7	31.3 ± 12*
Wisconsin card sorting test, categories	5.6 ± 0.7	5.5 ± 0.8
Wisconsin card sorting test, perseverative errors	1.8 ± 2.3	1.8 ± 2.4
Copying drawings	9.8 ± 2	9.6 ± 1.6
Copying drawings with landmarks	61.6 ± 5.8	63.1 ± 4.9

Values are means ± SD; *P < 0.01. *n* = 22. CEA: carotid endoarterectomy; CAS: carotid artery stenting.

**Table 4:** Psychiatric and behavioral assessments before and after CEA or CAS.

Hamilton depression rating scale (HDRS)	7.9 ± 5.7	8.4 ± 6.6
Beck depression inventory (BDI)	7.9 ± 6.7	6.6 ± 6.4
Hamilton anxiety rating scale (HAMA)	9.3 ± 5.6	9.2 ± 7.6

Values are means ± SD; *P < 0.01. *n* = 22. CEA: carotid endoarterectomy; CAS: carotid artery stenting.
